# Identification of a Chicken Anemia Virus Variant-Related *Gyrovirus* in Stray Cats in China, 2012

**DOI:** 10.1155/2014/313252

**Published:** 2014-02-13

**Authors:** Xinheng Zhang, Yuanjia Liu, Jun Ji, Feng Chen, Baoli Sun, Chunyi Xue, Jingyun Ma, Yingzuo Bi, Qingmei Xie

**Affiliations:** ^1^College of Animal Science, South China Agricultural University, Wushan Road, Tianhe District, Guangzhou, Guangdong 510642, China; ^2^College of Veterinary Medicine, South China Agricultural University, Guangzhou 510642, China; ^3^State Key Laboratory of Biocontrol, College of Life Sciences, Sun Yat-Sen University, Guangzhou 510006, China; ^4^Key Laboratory of Chicken Genetics, Breeding and Reproduction, Ministry of Agriculture, Guangzhou 510642, China

## Abstract

The chicken anemia virus (CAV), is a known member of the genus *Gyrovirus* and was first isolated from chickens in Japan in 1979. Some reports have also demonstrated that CAV can be identified in human stool specimens. In this study, a variant of CAV was detected using PCR with CAV-based primers in fecal samples of stray cats. The genome of CAV variant was sequenced and the results suggest that it could be a recombinant viral strain from parental CAV strains JQ690762 and AF311900. Recombination is an important evolutionary mechanism that contributes to genetic diversification. These findings indicate that CAV variant might have originated from CAV-infected chickens. The epidemiology and pathogenesis of this novel virus remains to be elucidated. This study underscores the importance of CAV surveillance and it presents the first evidence suggesting the possibility of CAV homologous recombination in cat.

## 1. Introduction

The chicken anemia virus (CAV) is a transmissible pathogen that infects young chickens, resulting in aplastic anemia, hemorrhages in the muscle and subcutaneous tissues, thymus atrophy, and severe immune suppression [1]. The infection can be propagated within the flock both vertically and horizontally. CAV is a member of the *Gyrovirus* genus, belonging to the family *Circoviridae *[2]. The CAV genome is comprised of single-stranded, circular DNA approximately 2.3 kb in length, with three partially overlapping open reading frames (ORFs), coding for the VP1, VP2, and VP3 proteins [3]. VP3, also named apoptin, is a proapoptotic protein. Infection with this virus, which is mainly transmitted through contaminated feathers, has been shown to trigger apoptosis in thymocytes and myeloid progenitor cells in young chickens [4]. Viral surveillance programs have isolated more than 50 CAV samples from different provinces in China during 2009–2012, and the epidemiological analysis suggested that CAVs were prevalent in China. We also analyzed the characteristic of one strain (GD-1-12), which was isolated in Southern China in 2012; the GD-1-12 isolate's genome at position 183 has a 21 nt deletion (TCCGTACAGGGGGGTACGTCA) in comparison with the genome (accession no. JQ690762) of the CAV isolate from human fecal samples. It is unclear if the 21 nt deletion is related to host specificity or pathogenicity [5]. In 2010, the CAV genome has been detected in breeder and commercial chicken flocks in South Korea [6]. The CAV (JQ690762) was also detected in pediatric fecal samples in Beijing, China (http://www.ncbi.nlm.nih.gov/nuccore/JQ690762/) [7]. In 2011, a novel human virus was identified on the surface of human skin and was designated human gyrovirus (HGyV), due to its homology with the chicken anemia virus [8]. In 2012, another gyrovirus species named GyV3 was detected in diarrhoea and normal faeces from Chilean children in the USA, which may reflect consumption of CAV-infected/vaccinated chickens due to the low sequence similarity with other gyroviruses [9]. Another gyrovirus (GyV4) was identified in human stool samples and in chicken meat sold for human consumption in Hong Kong in 2012 [10]. In the same year, four HGyV DNA samples were detected, three were from kidney transplant recipients and one was from an HIV-infected patient in Italy [11]. Human gyrovirus apoptin protein (VP3) has a similar subcellular distribution pattern and apoptotic function as its CAV homolog [12]. However, whether or not the first gyrovirus-carrying human was infected by eating CAV-infected/vaccinated chickens and the molecular mechanism of HGyV infection are still unclear and require further study. Cases of CAV recombination have been reported in chickens in China and the only recombination region is located in the coding region of VP1 [13, 14]. In this study, the first evidence of CAV homologous recombination in cats is explored in an attempt to elucidate the mechanism through which CAV infects various species.

## 2. Materials and Methods

### 2.1. Clinical Specimens

In order to identify circulating CAV viruses in cats in Southern China, 102 fecal samples were selected from two humane shelters located in Baiyun district (*n* = 40) and Conghua (*n* = 62) district of Guangzhou in 2012, where stray cats were most widespread in the south central area of Guangdong Province.

### 2.2. Extraction of Viral DNA

Viral DNA was extracted from 102 fecal samples using a commercial DNA extraction kit (QIAamp DNA Stool Mini Kit, QIAgen, Hilden, Germany) according to the manufacturer's instructions. The DNA was then quantitated and stored at −20°C until PCR was performed.

### 2.3. Virus Detection by PCR

The extracted DNA was screened by PCR. The primers were as sollows: JCP1: 5′CATCAACGGTGTTCAGGC3′ and JCP2: 5′CCTTGGAAGCGGATAGTCAT3′. Primers were designed by Primer Premier 5.0 to amplify 535 bp from the partial coding regions of CAV. The PCR amplification was performed in a total volume of 25 uL, including 12.5 uL of buffer I, 4 uL dNTPs, 0.5 uL of each primer, 6 uL distilled water, 1 uL DNA, and 0.5 uL LA Taq polymerase (TaKaRa, Biotechnology, Dalian, China). Amplification reactions were performed using the automated thermal cycler (Gene Amp PCR System 9700, Applied Biosystems, Foster City, CA, USA) with the following cycling profile: initial denaturation at 94°C for 4 min followed by 30 cycles of denaturation, annealing, and extension at 94°C for 30 s, 57°C for 30 s, and 72°C for 2 min, respectively, and a final extension step was carried out at 72°C for 10 min. The 535 bp reaction product was analyzed on 1% agarose gels. Negative controls were included in every three PCR reactions and one positive control was included in each set of reactions performed. The standardized PCR amplification yielded a distinct band of 535 bp in size as expected.

### 2.4. Amplification, Cloning, and Sequencing of the CAV Variant Genome

The DNA from the PCR-positive sample of CAV variant was further analyzed by two primers pairs to amplify the complete CAV variant genome. Primers KQ1F, 5′-CAATCACTCTATCGCTGTGT-3′ and KQ1R: 5′-TTCGTCCATCTTGACTTTCT-3′ and primers KQ2F: 5′-GGCTACTATTCCATC(A/T)CCATTCT-3′, and KQ2R: 5′-GCTCGTCTTGCCATCTTACA-3′, were designed to amplify 1778 bp and 831 bp fragments, respectively, covering the entire genome. The PCR amplification was carried out in 50 uL volume containing 25 uL buffer I, 16 uL dNTP, 0.5 uL of each primer, 13.5 uL distilled water, 1 uL of the DNA, and 0.5 uL LA Taq polymerase (TaKaRa, Biotechnology, Dalian, China). Amplification of the 831 bp region was carried out using the following PCR conditions: initial denaturation of 94°C for 4 min followed by 30 cycles of denaturation, annealing, and extension at 94°C for 30 s, 59°C for 30 s, and 72°C for 2 min, respectively, and a final extension step was carried out at 72°C for 10 min. Amplification of the 1778 bp region proceeded for 35 cycles as follows: 5 min at 94°C, 30 s at 94°C, 30 s at 58°C, 2 min 30 s at 72°C, and a final extension step was carried out at 72°C for 10 min. PCR amplification products were analyzed on 1% agarose gels stained with ethidium bromide. PCR products were purified using the Gel Band Purification Kit (Omega Bio-Tek, USA) and cloned into the pMD19-T vector (TaKaRa Bio Inc, Japan) followed by sequencing in triplicate using an ABI 3730 Sanger-based genetic analyzer (Carlsbad, CA, USA).

### 2.5. Sequence Alignment

The complete nucleotide sequence of CAV variant and reference sequences from different hosts from various countries were available from GenBank (Table 1). The DNA sequences and amino acid sequences were assembled using DNAStar (version 7; Madison, WI, USA) and Multiple sequence alignment was performed with the Clustal X (BioEdit version 7) program. Phylogenetic trees were constructed for genome sequences using the MEGA 5.1 program [15].

### 2.6. Sequence Recombination Analysis

Detection of potential recombinant sequences, identification of potential parental sequences, and localization of possible recombination break points were performed with the Recombination Detection Program (RDP4) v.4.1.3 [16]. Five different methods (RDP, Chimaera, MaxChi, Bootscan, and 3Seq) were used to detect recombination events in Cat-GyV. Meanwhile, in order to confirm the results, putative recombinant sequences and their parental strains were further analyzed with Simplot version 3.5.1 [17]. Evidence for networked relationships in CAV variant was examined using MEGA 5.1.

## 3. Results

### 3.1. Prevalence of CAV Variant

The survey data showed that the percentage of CAV positive samples was nearly 10% (10 of 102 fecal samples).

### 3.2. Molecular Characteristics and Phylogenetic Analysis of CAV Variant

**Figure 1:**
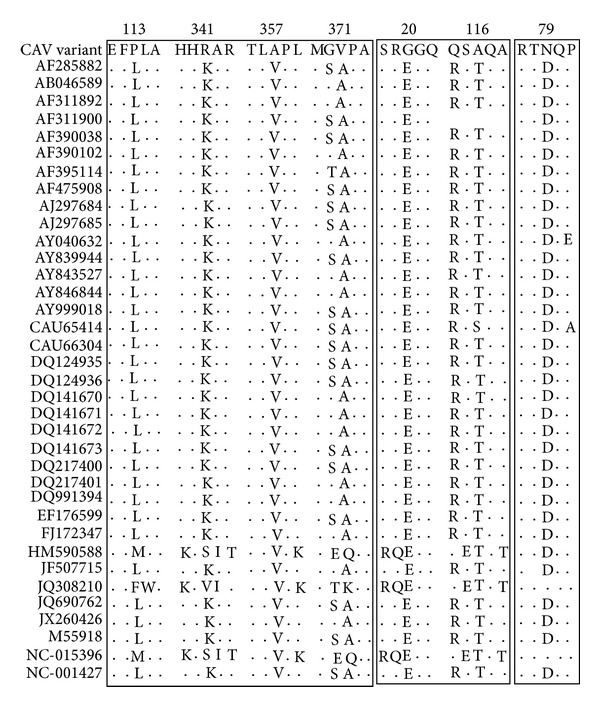
Multisequence alignment of deduced amino acids of the coding region at positions 113, 341, 357, 371 (VP1), 20, 116 (VP2), and 79 (VP3).

The complete genome sequence of CAV variant was submitted to GenBank, under the accession number KC414026. The CAV variant genome was 2,295 nt long, very close to the genome size (2,316 nt) of the CAV isolated from human fecal samples (accession no. JQ690762). Comparative analyses showed that CAV variant shared the greatest sequence identity (98.1%) with the CAV isolate from Japan (AH9410) and the least identity (38%) with the GyV3 isolate from the USA. The VP1, VP2, and VP3 genes of the CAV variant showed nucleotide variations of 1–63.1%, 0.5–51.9%, and 0.5–50.5%, respectively, among the 36 relevant sequences from GenBank. Previous studies have confirmed that the VP1 protein has the highest variability with a hypervariable region located from residues 139 to 151, while the amino acids 139 and 144 play vital roles in virus growth and spread, as VP1 residues Q139 and/or Q144 are associated with a decreased rate of spread of CIA-1 isolate [18]. In the CAV variant, VP1 residues 139 and 144 are lysine and glutamic acid, respectively, while in the two CAV isolates JQ690762 and AF311900, they are both glutamines. There were four unique amino acid mutations (L/M/F113P, K/S/V341R, V357A, and A371V) in VP1 of CAV variant, which were not previously reported (Figure 1). The VP1 residue 394 is a major genetic determinant of virulence, with glutamine (Q) and histidine (H) representing high and low pathogenicity, respectively [19, 20]. In the CAV variant, residue 394 was glutamine (Q) and was, therefore, highly pathogenic. There were two mutations (E20G, T/S116A) in VP2 and one mutation (D79N) in VP3 of CAV variant, which had not been previously observed (Figure 1). Phylogenetic analysis of the complete genome of CAV variant showed that CAV variant clustered to a clade with the CAV strains in chickens (Figure 4(a)).

### 3.3. Recombination Analysis

**Figure 2:**
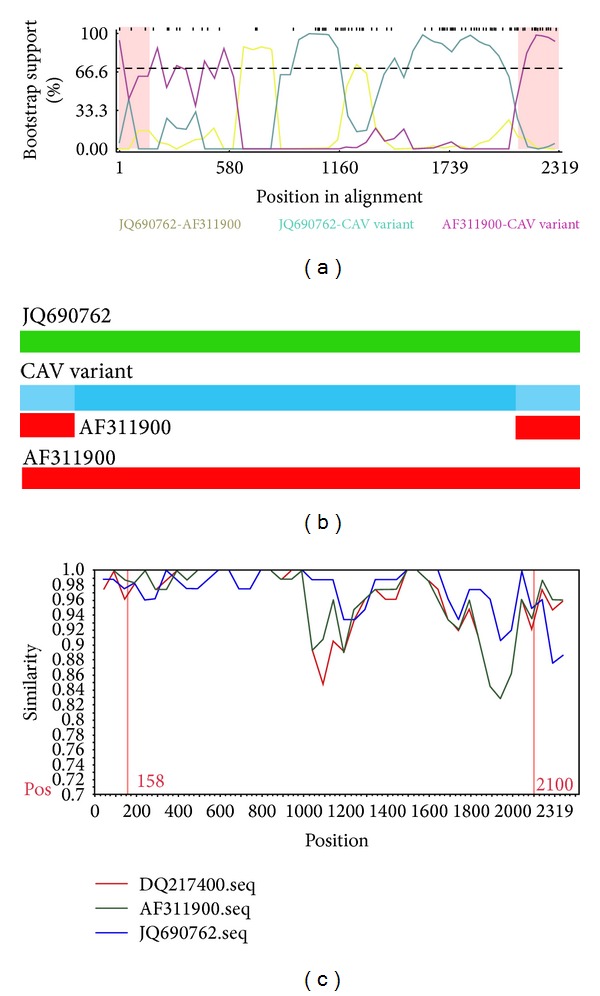
(a) and (b) Bootscan analysis of the recombinant, major parent, and the minor parent sequences. The analysis was based on a pairwise distance model with a window size of 200, step size of 50, and 1000 bootstrap replicates generated by the RDP4 program. (c) A comparison of the three CAV isolates: AF311900/chicken, JQ690762/human, and CAV variant/cat. The KC414026/CAV variant/cat was used as the query sequence. The DQ217400 was included as an outgroup. The *y*-axis gives the percentage of identity within a sliding window 80 bp wide centered on the position plotted, with a step size between plots of 50 bp.

**Figure 3:**
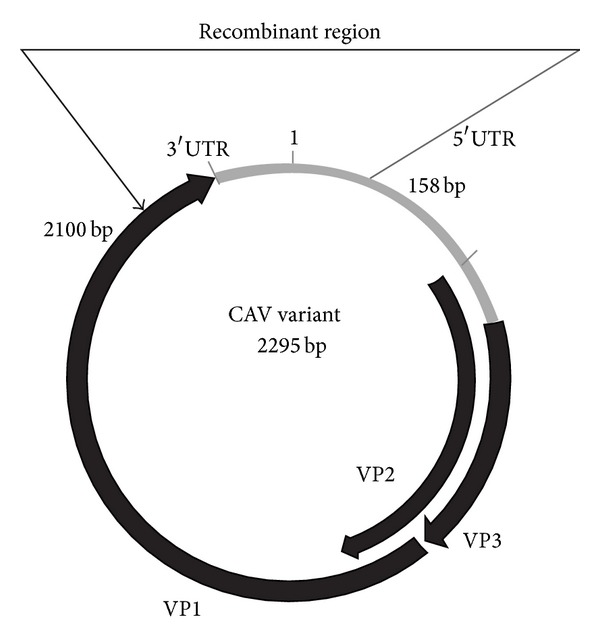
Map of CAV variant (accession no: KC414026) and the recombination breakpoints (beginning breakpoint at 2,100 and ending breakpoint at 158) in alignment with the CAV variant DNA nucleotide sequence.

**Figure 4:**
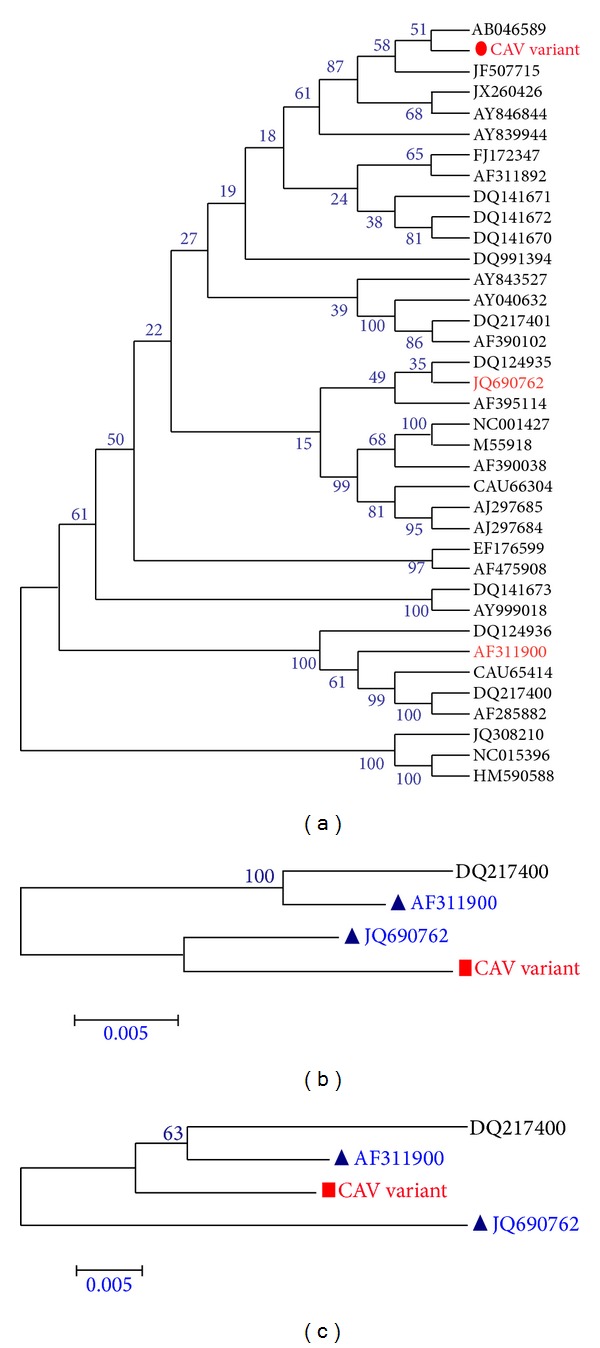
(a) Phylogenetic analysis of 37 CAV isolates in different species based on the genomic sequence. The three analyzed sequences (AF311900, JQ690762, and CAV variant) are indicated in red. The putative mosaic was indicated with “red dot.” (b) and (c), respectively, represent the nonrecombinant region (159-2,009) and the recombinant region (2,100-158). The putative recombination is shown with a “red square” and the putative parental lineages are shown with a “blue triangle.” The DQ217400 strain was used as an outgroup. The whole sequences were analyzed by using MEGA5.1 software with neighbor-joining (NJ) phylogenetic tree methods together with the novel sequence. Each tree was produced using a consensus of 1000 bootstrap replicates.

The above results suggested that CAV variant (accession no: KC414026) was a potential recombinant isolate between two CAV strains, namely, CAV strain AF311900 as the minor parent and CAV strain JQ690762 as the major parent (Figure 2) with the recombination breakpoints mapping to position 2,100 (beginning breakpoint) and 158 (ending breakpoint). The recombination region of CAV variant was located in the partial VP1 coding region, the entire 3′ untranslated region (UTR), and a portion of the 5′UTR (Figure 3). In order to identify if the two breakpoints are precise, we divided the CAV variant genome into two segments: the recombinant region from genomic nucleotide position 2,100-158 and the nonrecombinant region from genomic nucleotide positions 159-2,099. The phylogenetic tree revealed that the breakpoints delimit sequence regions with distinct phylogenies (Figures 4(b) and 4(c)). There was a recombination event in the nucleotide position from 2,100 to 158 of the CAV variant genome, which suggests that recombination could speed up the evolution of CAV.

## 4. Discussion

A CAV variant was isolated from stray cat fecal samples in China, and its genome was sequenced. The variant virus was found in 10% of samples. The CAV variant was highly pathogenic and had a very high sequence similarity with CAV. Davidson et al. reported that feathers contribute to the horizontal transmission of CAV, by carrying CAV either on their surface or within their feather pulp [21]. Although horizontal transmission is considered the most significant mode of CAV dissemination, the process is not well understood. Further analysis showed that CAV variant was a recombinant virus strain descended from CAV strain JQ690762 and CAV strain AF311900. Homologous recombination is considered rare in single-stranded circular DNA viruses and has not been reported for CAV in cats. Homologous recombination might result in the potential change in viral epidemiology. The results of this study indicate that CAV variant might have originated from CAV-infected chickens. It seems plausible that the stray cats were in contact with and/or ingested CAV-infected/vaccinated chickens. Similarly, humans could be similarly infected by contact with infected cats at the animal shelters. However, the pathogenesis of CAV variant in cats or chickens remains to be elucidated. So far, CAV has been identified in three different species including cats, chickens, and humans. This virus, first reported by Yuasa et al. (1978) [22] from contaminated vaccines in Japan, has a worldwide distribution. Phan et al. confirmed a high prevalence of CAV DNA in diarrhoea and normal faeces from Chilean children [9]. Chu et al. reported that the high rate of codetection of the three gyroviruses (CAV, AGV2/HGV and GyV3) in human specimens may indicate common dietary exposure to foods that contain all three viruses (e.g., chicken skin and meat) or it may imply some undefined dependency or codependency for successful proliferation [10]. As an immune suppression agent, CAV is often associated with secondary bacterial and viral infections. Whether human infection results in reduced immunity to other foreign agents and morbidity requires further study. It is important to understand the extent of sequence variability in CAV, in order to improve the management strategies to prevent CAV infections in chickens and to prevent the transmission from chickens to humans or cats. The epidemiology and pathogenesis of this virus in chickens and in cats require further investigation. Our reports also underscore the need to study and monitor circulating CAVs in China.

## Figures and Tables

**Table 1:** The GenBank accession numbers of full-length CAV genomes in isolates from different host species.

Accession number	Strain name	Host	Year	Country (area)
AB046589	AH9410	Chicken	2001	Japan
AF285882	SMSC-1	Chicken	2003	Malaysia
AF311892	98D02152	Chicken	2010	USA
AF311900	98D06073	Chicken	2010	USA
AF390038	3-1	Chicken	2003	Malaysia
AF390102	SMSC-1P60	Chicken	2003	Malaysia
AF395114	BD-3	Chicken	2004	Bangladesh
AF475908	—	Chicken	2002	China (Harbin)
AJ297684	Cux-1	Chicken	2000	Germany (Cuxhaven)
AY040632	3-1P60	Chicken	2003	Malaysia
AJ297685	Cux-1	Chicken	2000	Germany (Cuxhaven)
AY839944	LF4	Chicken	2004	China
AY843527	TJBD33	Silkies	2005	China
AY846844	TJBD40	Chicken	2004	China
AY999018	SD24	Chicken	2005	China
CAU65414	704	Chicken	1996	Australia
CAU66304	isolate 10	Chicken	1997	UK
DQ124935	AH6	Chicken	2005	China (Anhui)
DQ124936	AH4	Chicken	2005	China (Anhui)
DQ141670	SH11	Chicken	2005	China (Shanghai)
DQ141671	SH16	Chicken	2005	China (Shanghai)
DQ141672	HN9	Chicken	2005	China (Henan)
DQ141673	SD22	Chicken	2005	China (Shandong)
DQ217400	SMSC-1P9WT	Chicken	2005	Malaysia
DQ217401	SMSC-1P123WT	Chicken	2005	Malaysia
DQ991394	01-4201	Chicken	2007	USA
EF176599	C14	Chicken	2007	China
FJ172347	SDLY08	Broiler chicken	2008	China
HM590588	AGV2	Chicken	2011	Brazil
JF507715	CIAV89-69	Chicken	1991	South Korea
JQ308210	GyV3	Human	2011	USA
JQ690762	AGV2	Human	2012	China
JX260426	GD-1-12	Chicken	2012	China (Guangdong)
M55918	Cuxhaven-1	Chicken	2008	Netherlands
NC_001427	—	Chicken	2009	USA
JX310702	GyV4	Human and chicken	2012	Hong Kong
KC414026	CAV variant	Cat	2012	China (in this paper)
